# Study of a Mode Separation Due to Polarization Existing in a Cavity-Enhanced Absorption Spectroscopy

**DOI:** 10.3390/s21217101

**Published:** 2021-10-26

**Authors:** Shiyu Guan, Dingbo Chen, Huilin Cao, Zhongqi Tan

**Affiliations:** College of Advance Interdisciplinary Studies, National University of Defense Technology, Changsha 410073, China; guanshiyu14@nudt.edu.cn (S.G.); chendingbo15@nudt.edu.cn (D.C.); caohuilin20@nudt.edu.cn (H.C.)

**Keywords:** laser spectroscopy, cavity-enhanced absorption spectroscopy, resonant cavity, polarized light

## Abstract

A special phenomenon of resonance mode separation is observed during the study of a high sensitivity folded-cavity enhanced absorption spectroscopy for the measurement of trace gases. The phenomenon affects the measurement of gas absorption spectrum in the cavity. This resonant mode separation phenomenon of the resonant cavity is different from the resonant modes previously observed in linear-cavity enhanced absorption spectroscopy systems. To explore the mechanism of this phenomenon, a series of hypotheses are proposed. The most likely reason among these hypotheses is based on the different reflectance properties of the plane mirror at the fold of the cavity for S-polarized light and P-polarized light. Based on the matrix calculation method, the different reflectance and phase shift of the plane mirror for S-polarized light and P-polarized light are analyzed theoretically, and the results are in better agreement with the phenomena observed in the experiment. Finally, in order to eliminate the resonant mode separation phenomenon, line polarizers were added. By improving the system, the cavity enhanced absorption spectrum of residual water vapor in the cavity was successfully measured, and a minimum detectable absorption coefficient of *α*_min_ = 7.6 × 10^−9^ cm^−1^ can be obtained in a single laser scan of 10 s.

## 1. Introduction

With the development of laser technology, a series of novel high-sensitivity spectroscopy techniques have been proposed. Spectroscopies based on photoacoustic [1,2], intracavity absorption [3,4] and wavelength modulation [5,6], etc., have been progressing based on laser technology. Cavity-enhanced absorption spectroscopy (CEAS) arose in the 1990s [7], and in recent years it has been developing rapidly and has become an important part of laser spectroscopy. CEAS is now widely used in the fields of substance spectral detection, air pollution detection, and breath analysis [8,9]. Distinct to spectroscopy techniques, which enhance the effective absorption path by high-finesse optical cavities, such as off-axis incidence cavity-enhanced absorption spectroscopy [10], quartz-enhanced photoacoustic spectroscopy [11,12], cavity-enhanced optical external aberration molecular spectroscopy [13], and cavity ring-down spectroscopy (CRDS) [14,15,16]. Cavity-enhanced absorption spectroscopy refers specifically to the technique of obtaining the absorption spectrum by measuring the resonant transmission signal of a high finesse optical cavity.

In 1998, Engeln proposed the CEAS technique [7] as a modification of CRDS [16]. CEAS is essentially one of the direct absorption spectroscopy techniques and it takes advantage of a high-quality passive optical resonant cavity [17]. Since the laser satisfying the resonance condition can make multiple round trips in the passive cavity, the effective absorption path of the absorbing medium is enhanced exponentially over the limited optical path length [18]. Therefore, the measurement sensitivity of the CEAS technique is dramatically improved compared to the common direct absorption spectroscopy technique. In CEAS, the light intensity continuously transmitted by cavity resonances is recorded, rather than decaying [17]. CEAS does not require high detector performance and does not need a modulation device such as an optical switch (which is used in CRDS for fast signal shutdown). Therefore, the CEAS system is simple in structure and relatively low in cost.

In the application of high-finesse optical resonant cavity laser absorption spectroscopy, represented by CEAS and CRDS, some phenomena often occur in the absorption spectrum, such as the etalon effect and ripple effect [19,20,21]. These phenomena usually lead to degradation of the system’s performance and limit its application. In this paper, we designed and built a CEAS system based on a passive optical folded-resonant cavity. During the study of this system, an undesirable phenomenon of the resonant modes separation was observed. This is inconsistent with the longitudinal mode spacing determined by the free spectral range and has a negative impact on the measurement performance of the system. To explore the potential causes of this phenomenon, and assuming a correlation with the light polarization, an improved CEAS system which can adjust the light polarization is established. A method to prevent and eliminate this phenomenon is also proposed. Experimental results testify the relationship between the phenomenon and the light polarizations. Based on the matrix calculation methods, the different reflectance and phase shift of the plane mirror for S-polarized light and P-polarized light are analyzed theoretically. This could explain the main causes of the resonant mode separation phenomenon. These works are significant to guide the design of CEAS systems with fold-shaped cavity and to improve the system performance.

## 2. Experimental Setup and Measurement Principle

The cavity-enhanced absorption spectroscopy, whose principle has been described in several literatures [7,22,23,24], is similar to the direct absorption spectroscopy. By directly measuring the transmitted light intensity of the resonant cavity and combining the Beer-Lambert law of gas absorption, the concentration of the absorbed substance in the cavity is determined by Equation (1):

(1) $$I\left(v\right)=I_{0}\left(v\right)\exp\left[-\alpha \left(v\right)L\right],$$

where *I(v)* represents the transmitted light intensity; *v* represents the light frequency; *I*_0_*(v)* represents the incident light intensity; *α* denotes the absorption coefficient, which is related to the concentration of the absorbing substance, the intensity of the absorption lines, the line function, etc.; and *L* denotes the effective absorption path. The high finesse corresponds to the path length *L* enhancement induced by the cavity, which improves the detection sensitivity of system.

**Figure 1:**
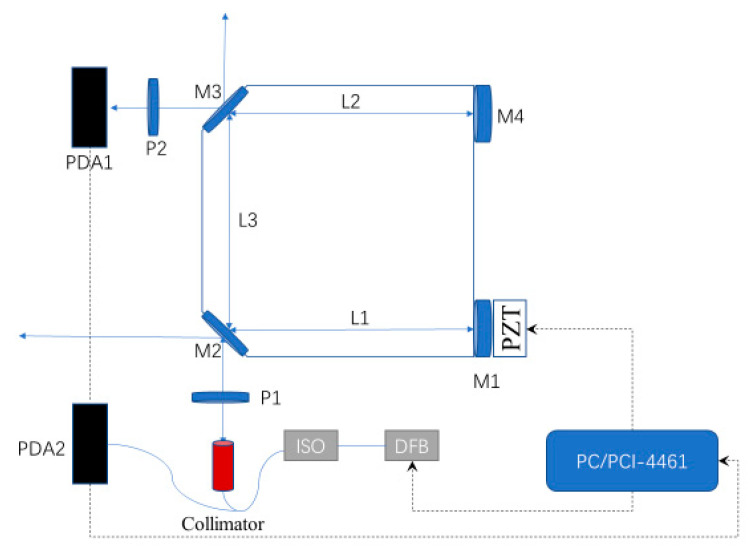
Structure diagram of a folded-cavity enhanced absorption spectroscopy experimental setup. M1–M4: mirrors; DFB: distributed feedback; PDA: photodetector; PZT: piezoelectric ceramic transducer; ISO: optical isolator; P1, P2: line polarizer.

As shown in Figure 1, the distributed feedback laser works under the control of high precision temperature driver and current driver. In this experimental setup, the light from DFB laser diode (SWLD-151210P22-09, Allwave Lasers, Xi’an, China) is coupled into a single-mode polarization maintaining (PM) fiber, and the pigtail output is connected with a 30 db fiber optical isolator. Then the optical isolator connected to a fiber coupler (50:50, 1550 nm Center Wavelength), and the two output ports of the fiber coupler are connected to an adjustable fiber collimator and a photo-detector PDA2 (PDA10CS-EC, Thorlabs, Newton, NJ, USA), respectively. The transmitted beam is transformed into a Gaussian beam by the collimator with adjustable optical waist radius and a divergence angle of about 0.074°, and its optical waist position can be adjusted at any distance from 0.3 m to infinity. The optical cavity has a special U-shaped configuration in which three arms form two folded angles of 45°. The cavity is made of highly stable glass ceramics for ultralow expansion coefficient, the length of L1 and L2 is 170 mm, and the length of L3 is 132 mm. The two end mirrors M1 and M4 are spherical mirrors with the same radius of curvature of 8 m, and the two folded mirrors M2 and M3 are plane mirrors. The ultra-low loss thin film coats in the central area of the mirrors possesses a diameter of 10 mm (R ≈ 99.99% at 1550 nm). The transmittance is less than 0.05% in its working band from 1500 to 1600 nm. A piezoelectric ceramic transducer (PZT) cavity length modulator is mounted on the end mirror M1 to adjust the cavity length in a tiny region. Finally, the cavity physical length is 472 mm. During the measurement of spectrum and optical path alignment, the PZT is driven by a triangular wave signal with an amplitude of 200 V and a frequency of 20 Hz generated by the driver circuit to modulate the cavity length.

In order to match the mode of resonant cavity with the incident laser, we simulate the intrinsic cavity mode parameters of the folded cavity in advance. By adjusting the parameters of the zoomable fiber collimator, the beam waist radius and beam waist distance of the incident Gaussian beam can be changed. Here, the parameters of the zoomable collimator are set to 0.8 mm optical waist radius and 0.3 m optical waist position. It is almost identical to the resonant cavity’s intrinsic cavity mode at the laser’s center frequency (1512 nm), implying a perfect mode match between the incident laser and the resonant cavity.

When the resonance occurs, the PDA1 (PDA10CS-EC, Thorlabs, Newton, NJ, USA) obtains parts of resonant light signal and sends the signal to the high-speed data acquisition card (NI-PCI-4461, National Instruments, Austin, TX, USA) in PC. The measurement program obtains the peak light intensity signal corresponding to the incident laser frequency as the cavity-enhanced spectrum signal. At the same time, the real-time intensity signal of the laser obtained by the PDA2 is also recorded by the acquisition card. The laser intensity signal is used as a reference signal for the cavity enhancement signal to eliminate the effects caused by incident laser power fluctuations. The absorption information of the absorbing medium in the cavity can be obtained by Equation (2) [22]:

(2) $$\alpha =\frac{1-R}{L}(\sqrt{\frac{I_{0}}{I}}-1),$$

Here, *α* represents the gas absorption coefficient; *c* represents the speed of light; *R* represents the effective reflectivity determined by the cavity decay of “empty cavity”; *L* represents the cavity length; *I*_0_ represents the transmitted light intensity without gas absorption; *I* represents the transmitted light intensity with gas absorption.

During a single laser scan, the operation temperature is constant, and a voltage ramp is fed to the current driver to achieve an accurate laser frequency scanning. The calibration of laser frequency is realized by a high-precision wavelength meter (WA-1500-NIR, EXFO Burleigh, Canada). After the wavelength meter calibration test, it is known that the DFB semiconductor laser can achieve continuous scanning in the range 1510–1512 nm when the temperature is controlled at 21 °C and the injection current is varied in the range of 40–120 mA. The PZT cavity length modulator can modulate the cavity length at each injected laser frequency to obtain resonance, at which point the spectral resolution of the cavity-enhanced absorption spectroscopy system will depend on the injection current modulation accuracy of the DFB semiconductor laser. This is distinct from classical cavity-enhanced absorption spectroscopy systems, whose spectral resolution is determined by the free spectral range of the resonant cavity. Thus, our system improves the spectral resolution from the free spectral range determined by the resonant cavity length (about 0.03 cm^−1^) to about 0.002 cm^−1^. The processing of simultaneous signal and the generation of control signal are realized by LabVIEW. Finally, the absorption spectrum after each laser scan were fitted based on the HITRAN database and the gas concentration was calculated from Equation (3):

(3) $$C=\frac{\alpha }{N_{L}\sigma },$$

where *C* represents the gas concentration; *α* represents the gas absorption coefficient; *N_L_* represents the molecular number density; and *σ* represents the gas absorption cross section obtained from the HITRAN database [25].

In addition, in order to verify our study on the resonant mode separation of the resonant cavity, which will be mentioned later, two line polarizers, P1 and P2, were added in our system as shown in the Figure 1. The two line polarizers can change and measure the polarization state of the incident light, respectively. In our system, P1 was used to change the polarization state of the laser light incident into the cavity and P2 was used to measure the polarization state of the resonant signal from the resonant cavity. Our theoretical analysis in Section 3 suggests that the phenomenon of resonant mode separation of the resonant cavity is related to the polarization state of light. The polarizers are added to verify the conclusion of the theoretical analysis, and to eliminate the effect of this phenomenon on the system performance and improve the system measurement sensitivity.

## 3. Results and Discussion

During optical path alignment and the measurement of spectrum, the PZT is driven by a triangular wave signal with an amplitude of 200 V and a frequency of 20 Hz is generated by the driver circuit to modulate the cavity length. The resonant mode separation phenomenon that we will discuss and analyze in this paper is also observed on the basis of this scanning signal.

### 3.1. Resonant Modes Separation Phenomenon and Discussion

**Figure 2:**
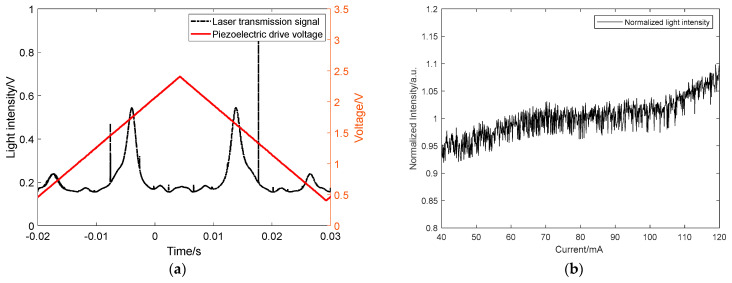
Resonant modes separation phenomenon and the corresponding spectral signal. (**a**) The resonant modes separation phenomenon; (**b**) The corresponding cavity-enhanced absorption spectrum.

As shown in Figure 2, two resonant transmission peaks with different resonant frequencies and linewidths appear in the transmission signal of the resonant cavity during a single scan period of the triangular wave. This phenomenon is different from that observed in our previous work [22,26,27,28], and it has a significant impact on the measurement of gas absorption spectrum. Therefore, the causes of the phenomenon should be carefully studied, and an elimination method needs to be proposed.

Considering the characteristics of the passive resonant cavity, we know that the two resonant modes separated from each other are not two consecutive longitudinal modes separated by a free spectral range (FSR). Combined with the features of the DFB semiconductor lasers we use, the good single-mode output of the laser also does not produce separated resonant modes at a fixed incident laser frequency. It should be noted that the optical resonant cavity we designed for the system has two 45° folded angles at the mirrors M2 and M3, and this modes separation phenomenon may be caused by the polarization state of the incident light.

The design of the folded-cavity allows for a longer optical path in a compact structure. It also prevents the direct reflection light at the incident mirror from being fed back to the laser, causing oscillations in the laser output pattern. The folded-cavity is a classical cavity structure in the application of optical feedback cavity-enhanced absorption spectroscopy (OF-CEAS) [29]. However, such a structure seems to make the cavity selective for the polarization of the incident laser. For a DFB semiconductor laser, the ideal polarization state of its output is linearly polarized light, and its light vector oscillates only in a defined direction. Usually, the polarized light whose polarization direction is perpendicular to the plane of incidence is called S-polarized light. In contrast, the polarized light whose polarization direction is parallel to the plane of incidence is called P-polarized light. The projection of the polarized light into two components, S-polarized light and P-polarized light, will be more beneficial to our understanding of the polarization state of the light wave.

Equations (4)–(7) are the Fresnel formulas for the reflection and transmission coefficients of light waves in the case of light waves incident on the interface of two media:

(4) $$r_{s}=-\frac{\sin(\theta_{i}-\theta_{t})}{\sin(\theta_{i}+\theta_{t})},$$

(5) $$t_{s}=\frac{2\cos\theta_{i}\sin\theta_{t}}{\sin(\theta_{i}+\theta_{t})},$$

(6) $$r_{p}=-\frac{\tan(\theta_{i}-\theta_{t})}{\tan(\theta_{i}+\theta_{t})},$$

(7) $$t_{p}=\frac{2\cos\theta_{i}\sin\theta_{t}}{\sin(\theta_{i}+\theta_{t})\cos(\theta_{i}-\theta_{t})},$$

where *r_s_* and *r_p_* denote the reflectance of the interface to S-polarized light and P-polarized light, respectively; *t_s_* and *t_p_* denote the transmittance of the interface to S-polarized light and P-polarized light, respectively; *θ_i_* denotes the angle of incidence; *θ_t_* denotes the angle of transmission.

**Figure 3:**
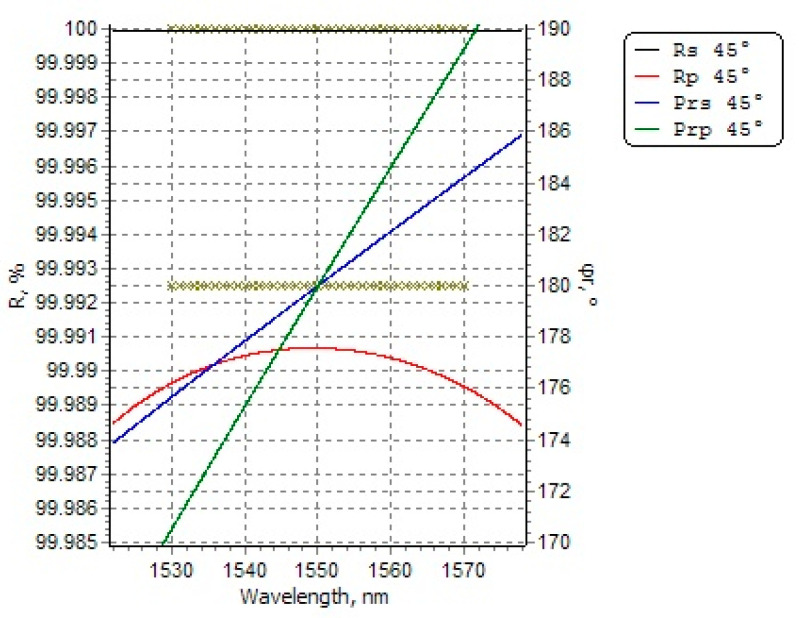
The simulation results of matrix calculation using the thin film software OptiLayer (version 8.85). The black line represents the reflection of S-polarized light at 45° oblique incidence; the red line represents the reflection of P-polarized light at 45° oblique incidence; the blue line the represents reflection phase of S-polarized light while the green line represents the reflection phase of P-polarized.

It is clear that for the case of non-perpendicular incidence (45° angle of incidence in our experimental setup), there is a significant difference between the reflection and transmission coefficients of S-polarized and P-polarized light. The case described above would be the simplest situation of a plane wave incident from one medium to another. As for the mirror coated with ultra-low loss thin film at our experimental setup, the ultralow loss thin film is a multilayer dielectric thin film made of successively spaced periodic stacks of high and low refractive index dielectrics (each layer has an optical thickness of λ/4). The ultra-low loss thin film provides enhanced reflectivity by using multibeam interference of light waves on all sides of the dielectric layer. The reflection and transmission coefficients as well as the phase shift of the light waves on the multilayer dielectric film need to be calculated based on the transmission matrix of the multilayer dielectric film. According to the thin film software OptiLayer’s simulation results, we can obtain the reflection and transmission coefficients as well as the reflection phase (corresponding to the center wavenumber of the thin film) of S-polarized light and P-polarized light on an ultralow loss thin film at the case of 45° oblique incidence, as shown in Figure 3.

According to the simulation results, it can be known that the reflection coefficient of S-polarized light is higher than that of P-polarized light at a 45° oblique incidence, and there will be some difference in the reflection phase of these two kinds of polarized light. Therefore, the specific cause of the resonance mode phenomenon can be analyzed based on the difference between S-polarized light and P-polarized light on the thin film.

Equations (8) and (9) are the formulas for resonant cavity finesse and transmission peak linewidth, respectively:

(8) $$F=\frac{\pi \sqrt{R}}{1-R},$$

(9) $$\delta v=\frac{c}{2\pi l}\times \frac{1-R}{\sqrt{R}},$$

Here, *F* denotes resonant cavity finesse; *R* denotes the effective reflectivity of the cavity mirror; *δ* denotes the linewidth of the resonant transmission peak; *c* denotes the speed of light; and *l* denotes the resonant cavity length.

It is known that the equivalent reflectivity of the mirror determines the finesse of the resonant cavity and the transmission peak linewidth. The higher the equivalent reflectivity, the higher the finesse of the cavity, and the narrower the corresponding resonant transmission peak linewidth. At the position of the two folded mirrors, since the reflection coefficients of S-polarized light and P-polarized light are different, the corresponding resonant cavity finesse will also differ. The cavity finesse of S-polarized light will be larger than that of P-polarized light, and its corresponding resonant transmission peak linewidth will be narrower than the latter, which also agrees with the separation of the two separated resonant transmission peaks observed in Figure 2a. 

Further, for a resonant cavity, resonance occurs only when the frequency and cavity length meet the standing wave condition shown in Equation (10), and the light in the cavity can be transmitted.

(10) $$\Delta \phi =\frac{2\pi }{\lambda }\times 2l\times n+2\left(\Delta \phi_{2}+\Delta \phi_{3}\right)+\Delta \phi_{1}+\Delta \phi_{4}=k\times 2\pi .$$

In Equation (10), *∆ϕ* denotes the phase difference of the laser in one round trip inside the cavity; *λ* denotes the laser wavelength; *l* denotes the resonant cavity length; *n* ≈ 1 denotes the refractive index of the gas in the cavity; ∆*ϕ*_1_–∆*ϕ*_4_ denote the phase shift of laser after reflection by mirrors M1–M4 respectively and *k* denotes an arbitrary integer. In the case of our experiments, the signal of the resonant transmission peak always corresponds to the same PZT driving voltage in each cavity length scan period due to the stability of the resonant cavity. However, the difference exists in reflection phase between S-polarized light and P-polarized light at the folded mirror. It means that even if the incident light is monochromatic, the effective optical paths *l* of its S-polarized light and P-polarized light inside the cavity are different, and the standing wave condition is satisfied differently. Therefore, due to the presence of the folded mirror, the S-polarized light component and the P-polarized light component of the same incident laser beam should correspond to different *l* in the standing wave condition, which also coincides the two relatively separated resonant transmission signals we observed earlier.

In summary, due to the special structure of the optical cavity and the reflective transmission nature of the multilayer dielectric high-reflective film for oblique incidence at 45° angle, the resonant cavity will separate the injected laser along the S-polarized direction and P-polarized direction, forming two stable, separated resonant modes with different finesse and resonant frequencies.

### 3.2. Experimental Results and Discussion

According to the previous theoretical analysis, the main reason for the resonant mode separation observed in the study is that the polarization direction of the incident light is not perpendicular to the plane of incidence. Both S-polarized light and P-polarized light exist in the cavity, respectively. To verify the correctness of the above theoretical analysis, experiments are designed to verify the polarization state of the resonant light signal to determine whether the two separated resonant transmission peaks belong to two mutually perpendicular polarization directions. It is also necessary to verify the relationship between these two separated resonant transmission peaks and the polarization direction of the incident light, which is probable to rotate within the resonant cavity. Therefore, as mentioned earlier in the introduction of the experimental setup, two linear polarizers were added between the collimator and the resonant cavity, and between the resonant cavity and the detector, respectively. The effect of different polarization directions on the resonant light signal can be observed visually by turning the polarizer direction. The linear polarizer allows the transmitted light to only have the same polarization component as the polarizer. It is able to analysis the polarization direction of the resonant transmitted light qualitatively and control the polarization direction of the incident light quantitatively.

**Figure 4:**
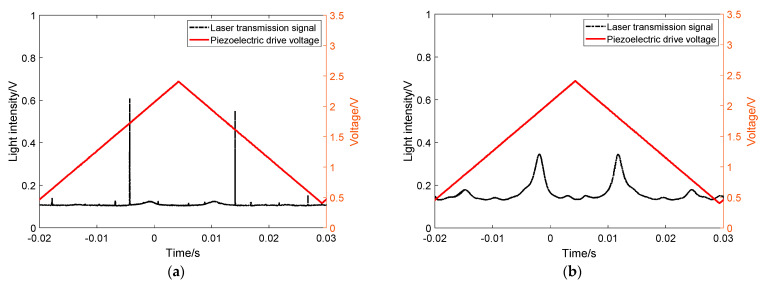
Resonant light signal by turning the line polarizer between mirror M3 and detector PDA1. (**a**) The signal of S-Polarized direction; (**b**) the signal of P-Polarized direction.

First, a line polarizer is set between mirror M3 and the photodetector, and when the polarizer is turned, the signal of the resonant light is shown in Figure 4.

As shown in Figure 4a when the line polarizer is rotated to the S-polarization direction, only the resonant light signal with the narrower linewidth of the two resonant light transmissions is present in the resonant light signal; Figure 4b on the contrary, when turning the line polarizer to the P-polarization direction, the resonant light signal is only the resonant light signal with the wider line width of the two resonant transmission signals. This result indicates that the two separated resonant modes belong to the mutually perpendicular S-polarized light and P-polarized light and exist independently of each other.

**Figure 5:**
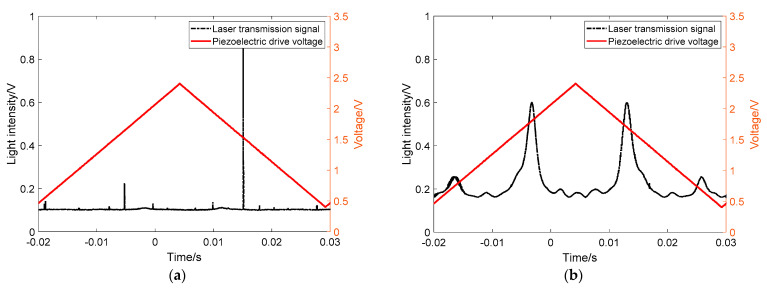
Resonant light signal by turning the line polarizer between mirror M2 and the collimator. (**a**) The signal of S-Polarized direction; (**b**) the signal of P-Polarized direction.

Then, the line polarizer P2 was removed between the folded mirror and the photodetector, the line polarizer P1 at the fiber collimator and the incident mirror M2 was added, and the polarizer was rotated in the same way. The experimental phenomenon is shown in Figure 5.

Consistent with the phenomenon of the previous experimental results, when turning the line polarizer to the S-polarization direction, only the resonant light transmission signal with narrower line width was observed; on the contrary, when turning the line polarizer to the P-polarization direction, only the resonant light transmission signal with wider line width appeared. The two experimental results can strongly certify the accuracy of our previous analysis. The two separated resonant modes belong to the mutually perpendicular S-polarization and P-polarization directions respectively, and they form their own resonant modes in the resonant cavity independently of each other.

It should be noted that the laser linewidth is about 3 MHz, while the linewidth of resonant cavity in S-polarization is about 19.41 kHz corresponding to the decay time of 8.2 us. Because the wide linewidth laser is injected into the narrow linewidth resonant cavity, the resonant transmission signal is influenced by current noise, etc. Therefore, in Figure 2a and Figure 5a, one of the S-polarized modes have less intensity. However, for the P-polarized modes, both the P-polarized modes’ intensity look identical due to its lower finesse and broader linewidth.

**Figure 6:**
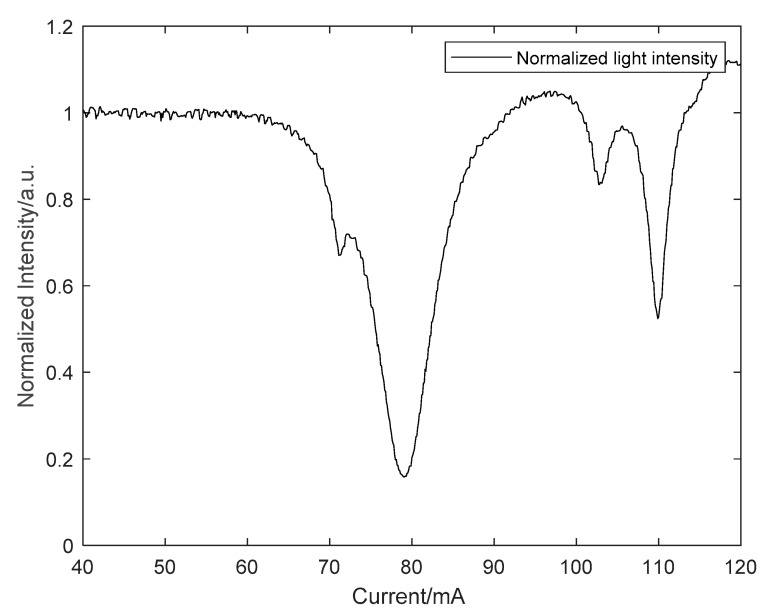
The cavity-enhanced absorption spectrum in the situation of the S-polarization direction.

Finally, two line-polarizers, P1 and P2, were placed into the positions illustrated in Figure 1, and the measurement of the residual air inside the cavity shown earlier was repeated. This experiment can test the feasibility of the system improvement scheme, and the performance of the improved system. The experimental results are shown in Figure 6.

According to the results shown in Figure 6, four absorption peaks can be clearly observed in the resonant transmitted light intensity signal measured in the S-polarization direction. In contrast, these absorption peaks cannot be perceived in the normalized light intensity signal shown in Figure 2. By comparing with the HITRAN database, all the absorption peaks we measured are absorption lines of water vapor. According to the decay time of 8.2 µs, the empty cavity loss is 191.8 ppm, and the effective reflectivity *R* is 0.9998. The standard deviation of the fitting residuals is 3.7 × 10^−3^, and a minimum detectable absorption coefficient of *α*_min_ = 7.6 × 10^−9^ cm^−1^ can be obtained by Equation (2) in a single laser scan of 10 s. The experimental results indicate that the improvement of the system effectively improves the sensitivity of cavity-enhanced absorption spectroscopy for the detection of trace gases. It also illustrates the difference in the finesse between the S-polarization and P-polarization directions, with the S-polarization direction having a higher finesse.

At last, a series of experiments were designed to prove the accuracy of the previous theory. The experimental results show that for the resonant cavity in our system, the incident light in the S-polarization and P-polarization directions will resonate at different cavity lengths, corresponding to different finesse and resonant transmission linewidths. As for the cavity-enhanced absorption spectroscopy, when the resonant cavity is a folded structure, the resonant mode separation phenomenon caused by the polarization direction needs to be avoided. In fact, it is necessary to control the polarization direction of the incident light to obtain a higher resonant cavity finesse (S-polarization direction in our device), which is important to guide the design of cavity-enhanced absorption spectroscopy and improve the system performance.

For other aspects, the phenomenon of resonant mode separation due to polarization may be of some positive significance. The concentration of trace gases can be accurately detected using S-polarized light with high finesse. Whereas, in the case that gas concentration is high, the intensity of S-polarized resonant light decreases extremely due to gas absorption. Weak signals will affect the signal-to-noise ratio of the system and influence the results of the CEAS measurements. While the P-polarized light can be used to measure high concentrations of gases due to its lower finesse. It can effectively broaden the detectable gas concentration range of gas detection sensors. The phenomenon is also instructive for thin film design studies. The reflectance of the film to S-polarized light and P-polarized light can be measured separately using CRDS. What is more, using the FSR of the cavity and the standing wave condition of Equation (10), the reflected phase delay between S-polarized light and P-polarized light can be obtained.

## 4. Conclusions

In conclusion, we have presented a near-infrared CEAS system with good spectral resolution. For the phenomenon of resonant mode separation observed in the experiments, theoretical and experimental studies were conducted. The mechanism of the resonant mode separation phenomenon is presented and calculated theoretically. The results show that the mirrors with a multilayer dielectric ultra-low loss thin film can separate the S-polarized light from the P-polarized light. Due to the difference of cavity finesse in different polarization directions, the polarization direction light with lower cavity finesse will limit the detection sensitivity of the system. The addition of line polarizers allowed to improve the system, namely by preventing and eliminating the resonant mode separation phenomenon observed, leading to the observation of several absorption lines of residual water vapor in the cavity. A minimum detectable absorption coefficient of *α*_min_ = 7.6 × 10^−9^ cm^−1^ can be obtained in a single laser scan of 10 s. This trace gas detection sensor has a compact structure and potential to provide stable performance in breath applications according to our previous work [29]. Potential applications of this phenomenon are also proposed in our paper.

## Data Availability

The data that support the findings of this study are available from the corresponding authors upon reasonable request.
